# Predictors of Duration and Treatment Failure of Severe Pneumonia in Hospitalized Young Nepalese Children

**DOI:** 10.1371/journal.pone.0122052

**Published:** 2015-03-23

**Authors:** Sudha Basnet, Arun Sharma, Maria Mathisen, Prakash Sunder Shrestha, Ram Kumar Ghimire, Dhiraj Man Shrestha, Palle Valentiner-Branth, Halvor Sommerfelt, Tor A. Strand

**Affiliations:** 1 Child Health Department, Institute of Medicine, Tribhuvan University, Kathmandu, Nepal; 2 Centre for International Health and Centre for Intervention Science in Maternal and Child health, University of Bergen, Bergen, Norway; 3 Department of Microbiology and Infection Control, University Hospital of North Norway, Tromso, Norway; 4 Department of Radiology, Institute of Medicine, Tribhuvan University, Kathmandu, Nepal; 5 Department of Radiology, Kathmandu Medical College and Teaching Hospital, Kathmandu University, Nepal; 6 Department of Infectious Disease Epidemiology, Division of National Health Surveillance & Research, Statens Serum Institut, Copenhagen, Denmark; 7 Department of International Public Health, the Norwegian Institute of Public Health, Oslo, Norway; 8 Division of Medical Services, Innlandet Hospital Trust, Lillehammer, Norway; UCL Institute of Child Health, University College London, UNITED KINGDOM

## Abstract

**Background:**

Pneumonia in young children is still the most frequent cause of death in developing countries. We aimed to identify predictors for recovery and treatment failure in children hospitalized with severe pneumonia.

**Methods:**

We enrolled 610 Nepalese children, aged 2 – 35 months from February 2006 to June 2008. Study participants were provided with standard treatment for pneumonia and followed up until discharge. Three multiple regression models representing clinical variables, clinical and radiological combined and all variables, including C-reactive protein (CRP) and viral etiology were used to assess the associations.

**Results:**

The median age of study participants was 6 months with 493 (82%) infants and 367 (61%) males. The median time (IQR) till recovery was 49 (31, 87) hours and treatment failure was experienced by 209 (35%) of the children. Younger age, hypoxia on admission and radiographic pneumonia were independent predictors for both prolonged recovery and risk of treatment failure. While wasting and presence of any danger sign also predicted slower recovery, Parainfluenza type 1 isolated from the nasopharynx was associated with earlier resolution of illness. Gender, being breastfed, stunting, high fever, elevated CRP, presence of other viruses and supplementation with oral zinc did not show any significant association with these outcomes.

**Conclusion:**

Age, hypoxia and consolidation on chest radiograph were significant predictors for time till recovery and treatment failure in children with severe pneumonia. While chest radiograph is not always needed, detection and treatment of hypoxia is a crucial step to guide the management of hospitalized children with pneumonia.

## Introduction

Pneumonia is an important cause of illness and leading cause of death in young children in developing countries [1]. More than 99% of pneumonia deaths occur in low- and middle-income countries (LMICs) [2]. The recent estimate is a median incidence of 0.22 episodes per child year (e/cy) with severe pneumonia contributing to 11.5% in LMICs [3]. In Nepal the estimated incidence of pneumonia is 0.24 e/cy with 11.5% severe episodes in children under 5 years of age [3]. While several preventable risk factors for severe pneumonia in developing countries have been identified [4] there is limited data on predictors of illness duration. Because the diagnosis of severe pneumonia in low income settings to a large extent relies on clinical criteria, it is important to identify objective clinical signs at baseline that may guide subsequent management of a severe pneumonia episode. The World Health Organization (WHO) uses evidence based clinical guidelines for the care of sick children in hospitals of resource poor settings [5,6]. The aim of this study is to identify predictors for duration and treatment failure of WHO-defined severe pneumonia in hospitalized young children in Nepal.

## Methods

The study participants were recruited into a clinical trial [7] of zinc as adjuvant therapy to standard treatment of an acute episode of severe pneumonia, defined using WHO guidelines [8] and registered at clinicaltrials.gov as NCT00252304. Clearances were obtained from the ethics board of the Institute of Medicine, Tribhuvan University and Nepal Health Research Council, Kathmandu. We enrolled 610 children, 2–35 months of age from February 2006 to June 2008 at the Kanti Children’s referral hospital in Kathmandu, Nepal. The Kathmandu valley lies at an altitude of approximately 1400 meters above sea level.

Eligible children presenting to the Kanti Children’s hospital Emergency or Outpatient departments were screened for enrollment by trained physicians and first assessed for hypoxemia using a pulse oximeter (Nellcor Puritan Bennett NPB-40, Pleasanton, CA, USA) with a pediatric sensor (Nellcor Pedichek D-YSPD) and for presence of wheezing. Oxygen saturation (SpO_2_) was recorded twice after stabilization of the reading for one minute. The higher of the two readings was used. For children with SpO_2_ <90%, oxygen was provided prior to further evaluation. This oxygen saturation of <90% was the threshold used to define hypoxia based on WHO guidelines [8]. Children with wheezing were given up to three doses of nebulized Salbutamol 15 minutes apart, reassessed and excluded if lower chest indrawing (LCI) disappeared. History of illness was recorded and the findings of the physical examination were captured in a standardized form. Children were weighed using an Electronic Scale 890 (SECA, Hamburg, Germany) to the nearest 100 grams. Height in children 2 years of age or older was measured using a standard wooden height measuring board, while recumbent length in children less than 2 years old was measured using an infantometer, both to the nearest 0.1cm. Length-for-age and weight-for-length z scores were calculated using the 2006 WHO Child Growth Standards when these parameters were below-2z, the children were considered to be stunted or wasted, respectively. We measured hemoglobin concentrations using Hemocue (Ångelholm, Sweden). Chest x-rays (CXR) were taken in all children, not only to identify infiltrations, but also to detect pleural effusion, pneumothorax or suspected heart disease, which would make a child ineligible for the study. Children with recurrent wheezing (defined as >3 episodes over the past 6 months and on treatment with bronchodilators), disappearance of lower chest indrawing (LCI) after nebulized Salbutamol, severe wasting (weight-for-length <-3z), severe anemia (hemoglobin <7 g/dL), heart disease, documented tuberculosis, concomitant diarrhea with dehydration and those with severe illness requiring special care or surgical intervention were also excluded. We used standardized WHO criteria to identify radiographic pneumonia [9]. The CXRs were interpreted independently by two radiologists blinded to clinical data, classifying findings as end-point consolidation, other infiltrates or normal. If the two radiologists did not reach an agreement on consolidation, a second round of interpretation was carried out to arrive at a consensus. Informed consent was obtained for eligible children. Parents who could read and write signed the consent statement after they had read a written statement in the local Nepali language. For parents who were unable to read/write, verbal informed consent was obtained in the presence of a witness, whose name and signature was recorded in a register along with the child serial number of that particular participant. This procedure was approved by the ethics committee for the study. After obtaining consent, blood was collected and the first dose of intravenous antibiotics administered. This was followed by collection of nasopharyngeal aspirates for identification of 7 respiratory viruses, namely respiratory syncytial (RSV), Influenza A and B, parainfluenza (PIV) type 1, 2, and 3, and human metapneumovirus, using a commercially available multiplex reverse transcription polymerase chain reaction (PCR) assay. Details of the sampling technique and analyses have been described elsewhere [10].

### Definitions

Acute episode of severe pneumonia—A child presenting with cough (duration <14 days) and/or difficult breathing of </ = 72 hours duration with LCI.

Time till cessation of severe pneumonia—The period starting from enrollment to the beginning of a 24-hour consecutive period of absence of LCI, of hypoxia and of any danger signs.

Treatment failure—A requirement for a change in antibiotics to second line therapy in those with failure to improve, development of complications such as empyema/pneumothorax requiring surgical intervention or admission to the intensive care unit for ventilator and/or inotropic support.

Failure to improve—Persistence of LCI or of any danger signs present at enrollment despite 48 hours of treatment or appearance of new danger signs or hypoxia with deterioration of a patient’s clinical status any time after initiation of treatment.

Clinical improvement—Absence of danger signs, of hypoxia for a consecutive 24-hour, and of LCI for a 48-hour period.

Duration of hypoxia—The period from enrolment with SpO_2_ < 90% on pulse oximetry to the beginning of a 24 hour consecutive period of SpO_2_>90%, when breathing unassisted in room air.

Enrolled children were admitted to the hospital and monitored at 8 hourly intervals by study physicians until discharge. Benzyl penicillin (50,000 units/kg IV every 6 hourly) and Gentamicin (7.5 mg/kg IV once daily) were given till clinical improvement, following which children were discharged with advice to continue oral Amoxicillin to complete treatment for a total duration of 10 days. In addition, each participant received daily supplements (dispersible tablets with either zinc or placebo) during their stay in the hospital and up to a maximum of 14 days [7].

Antibiotics were changed to Cefotaxime in children who did not improve. A decision to change antibiotics was made only after consultation with senior pediatricians involved in the study. For children unable to eat/drink or breast feed, intravenous fluids were initiated. Humidified oxygen was given to children with documented hypoxia and discontinued when they were no longer hypoxic. The absence of hypoxia was confirmed after a second reading taken 30 minutes later.

### Statistical Analyses

Data were analyzed using Stata version 13 (Stata Corp, College Station, TX). We fitted multiple regression models using clinical (Model 1), clinical and radiological (Model 2) and all (Model 3) variables. Age was used as a categorical variable. Predictors of time till cessation of a severe pneumonia episode were identified in Cox proportional hazards models with the “exactp” option to handle ties. For treatment failure, we used logistic regression. Outcomes were coded such that Hazard ratios (HR) <1 for time till recovery indicates slower resolution of illness and Odds ratios (OR) > 1 increased odds of treatment failure. Initially we assessed the crude associations of relevant independent variables with the selected outcomes. Variables with p< 0.25 were included in the multivariable models and those variables which were still significant, i.e. being associated with the outcome with a p-value of < 0. 05 retained in the model. In these models we included the other variables one at a time and kept if significant. This manual stepwise approach were outlined by Hosmer and Lemeshow [11].

We tested the goodness-of-fit of the models by the method suggested by Hosmer and Lemeshow [11] and for logistic regression by calculating the dfbetas and hat statistics. We assessed the assumptions for the cox models using tests of specification (plotting of Schoenfeld residuals) and goodness of fit.

## Results

We enrolled 610 children meeting criteria for an acute episode of severe pneumonia. We excluded from the analyses 11 children with heart disease and 1 child with chronic cough discovered after inclusion. During the study, 4 of the 7 children transferred to the pediatric intensive care unit died, 9 (1.5%) parents withdrew consent and 2 were diagnosed with tuberculosis. Demographic, clinical and laboratory characteristics of study participants are outlined in Table 1. The median age of the 598 children was 6[Interquartile range (IQR) 3–10] months, 61% were boys and 82% infants (<12 months). Mean respiratory rate [Standard deviation (SD)] was 64 (12), 62% were hypoxic and 49% had a danger sign, while 10% had 3 danger signs. On chest auscultation, 82% of the children had wheezing and 92% had crepitations. C-reactive protein (CRP)> 40mg/L was detected in 30%, 13% had CRP> 80mg/L. At least one virus was isolated from 29% of the nasopharyngeal specimens; RSV being the most frequent. Radiographic pneumonia, defined as endpoint consolidation on chest X-ray, was detected in 164 of 457 (36%) chest radiographs available for interpretation.

**Table 1:** Baseline Characteristics of children ages 2–35 months with WHO defined severe pneumonia.

Background Characteristics	N	Value
Median age in months (IQR)	598	6 (3, 10)
Age categories		
• 2–6 months (%)	352	58.9
• 7–11 months (%)	141	23.5
• 12–23 months (%)	90	15.1
• 24–35months (%)	15	2.5
Boys (%)	598	367 (61)
Breastfed (%)	598	571 (96)
Wasted (Weight for height/length Z score < -2) (%)^#^	594	157 (26)
Stunted (Height for age Z score <- 2) (%)^#^	598	50 (8)
Underweight (Weight for age Z score <- 2) (%) ^#^	598	102 (17)
Mean age of mother (SD)	586	24.4 (4.1)
Illiterate mother (%)^##^	586	154 (26)
Illiterate father (%)^##^	588	38 (7)
Unemployed mother (%)**	584	418 (72)
Unemployed father (%)**	580	19 (3)
**Clinical Characteristics**		
Mean Respiratory rate as breaths per minute (SD)		
• 2–11 months	493	65(12)
• 12–35 months	105	61(12)
Hypoxia (SpO_2_ < 90%) at enrolment (%)	598	373 (62)
Febrile (Axillary temperature > 38.5°C) (%)	598	92 (15)
Danger signs (%)		
-Nasal flaring	597	232 (39)
-Grunting	598	131 (22)
-Head nodding	598	138 (23)
-Cyanosis	598	3 (0.5)
• Any one danger sign	598	294 (49)
• Presence of 3 danger signs	598	57 (10)
• Presence of 2 danger signs	598	96 (16)
• Presence of 1 danger sign	598	141 (24)
Wheezing (%)	598	492 (82)
Crepitations (%)	598	549 (92)
Laboratory Characteristics		
Mean CRP in mg/L (SD)	572	37.9 (51.2)
-CRP > 40mg/L	572	170 (30)
-CRP > 80mg/L	572	75 (13)
Nasopharyngeal aspirate positive for any virus (%)	596	175 (29)*
-Respiratory syncytial virus (%)	596	79 (13)
-Parainfluenza type 1 (%)	596	23 (4)
-Parainfluenza type 2 (%)	596	5 (1)
-Parainfluenza type 3 (%)	596	24 (4)
-Influenza A (%)	596	24 (4)
-Influenza B (%)	596	17 (2)
-Human metapneumovirus (%)	596	9 (1)
**Radiographic Pneumonia (%)**	457	164 (36)
**Supplemented with zinc (%)**	598	299 (50)

^#^ Calculated using WHO Growth standards.

*6 children were positive for 2 viruses simultaneously.

^##^ No schooling with inability to read part or whole of a sentence.

** No work/housework.

Median time (IQR) till recovery, using predefined criteria, was 49 (31, 87) hours while time till discharge (IQR) was 97 (83, 135). Treatment failure occurred in 209 of the 594 children (35%).

There was a near linear relationship between age and both outcomes, i.e. time till recovery and the risk of treatment failure (data not shown). An increment in age by one month was associated with an HR of 1.04 (95% CI: 1.03–1.06) for time till recovery and the OR for treatment failure was 0.93 (95% CI: 0.90–0.96).

Wasting (HR 0.79 95% CI: 0.65, 0.96), hypoxia (HR 0.62 95% CI: 0.51, 0.74) and presence of any danger sign (HR 0.76 95% CI: 0.64, 0.91) were independently associated with slower recovery (Table 2). These associations did not change substantially with the addition of other variables (Models 2 & 3). Radiographic pneumonia was also a significant predictor of delayed recovery (HR O.58 95% CI: 0.47, 0.72). Presence of Parainfluenza type 1 (PIV 1) in the nasopharynx was associated with earlier recovery (HR 2.46 95%CI 1.48, 4.09). Gender, being breastfed, stunting, high fever, CRP> 40 and >80 mg/L, presence of other viruses and supplementation with oral zinc did not show any significant association with time till recovery (Table 2).

**Table 2:** Predictors of Time till Recovery of illness episode in children 2–35 months hospitalized with WHO defined Severe Pneumonia.

Variables	Crude Hazard Ratio*	Adjusted Hazard Ratio* (number of observations)		
**Age in months**	1.04 (1.03, 1.06) < 0.001			
**Age categories**		**Model 1 (594)**	**Model 2 (455)**	**Model 3 (454)**
• 2–6 months	1.00	1.00	1.00	1.00
• 7–11 months	1.43 (1.17, 1.75) < 0.001	1.49 (1.21, 1.83) < 0.001	1.53 (1.22, 1.94) < 0.001	1.22, 1.94) < 0.001
• 12–23 months	1.55 (1.21, 1.97) < 0.001	1.68 (1.30, 2.16) < 0.001	1.46 (1.09, 1.97) 0.012	1.09, 1.97) 0.012
• 24–35months	3.39 (2.01, 5.71) < 0.001	4.22 (2.46, 7.25) < 0.001	3.33 (1.77, 6.27) < 0.001	3.33 (1.77, 6.27) < 0.001
**Gender**	1.11 (0.94, 1.31) 0.223			
**Breastfed**	0.97 (0.64, 1.47) 0.882			
**Wasting** (< -2 Weight for height/length)	0.76 (0.63, 0.91) 0.004	0.79 (0.65, 0.96) 0.017	0.79 (0.64, 0.98) 0.030	0.78 (0.63, 0.96) 0.022
**Stunting** (< -2 Height/length for age)	0.84 (0.62, 1.14) 0.261			
**Hypoxia** (SpO2 < 90%)	0.64 (0.54, 0.76) < 0.001	0.62 (0.51, 0.74) <0.001	0.71 (0.58, 0.88) 0.001	0.72 (0.58, 0.89) 0.002
**Febrile** (Axillary temperature > 38.5°C)	1.14 (0.91, 1.43) 0.248			
**Any danger sign**	0.69 (0.59, 0.82) <0.001	0.76 (0.64, 0.91) 0.003	0.74 (0.60, 0.90) 0.003	0.76 (0.62, 0.92) 0.006
**Radiographic pneumonia**	0.56 (0.46, 0.69) <0.001		0.58 (0.47, 0.72) <0.001	0.55 (0.45, 0.68) <0.001
**C-Reactive Protein (CRP)** > 40 mg/L	1.01 (0.84, 1.21) 0.918			
**Nasopharyngeal aspirate positive for virus**				
• Respiratory syncytial virus (RSV)	(0.62, 0.99) 0.049			
• Influenza A	0.78 (0.52, 1.19) 0.252			
• Influenza B	(0.49, 1.33) 0.399			
• Parainfluenza type 1 (PIV 1)	1.75 (1.14, 2.69) 0.010			2.46 (1.48, 4.09) < 0.001
• Parainfluenza type 2 (PIV 2)	(0.40, 2.36) 0.957			
• Parainfluenza type 3 (PIV 3)	(0.96, 2.17) 0.082			
• Human metapneumovirus (hMPV)	0.64 (0.32, 1.28) 0.203			
**Supplemented with zinc**	1.11 (0.94, 1.31) 0.221			

*Hazard ratios (95% CI) and P-value calculated using Cox Regression with exact p option. Hazard ratios <1 for time till recovery indicates slower resolution of illness.

Results of multiple regressions with P-value > 0.05 not shown in the table.

Model 1 (Clinical variables): Adjusted for gender, breastfed, febrile and treatment with zinc.

Model 2 (Clinical + Radiographic pneumonia): Adjusted for gender, breastfed, febrile and treatment with zinc.

Model 3 (All variables): Adjusted for gender, breastfed, febrile, CRP, nasopharyngeal aspirate positive for RSV, influenza A and B, PIV 2 and 3 and hMPV and treatment with zinc.

When estimating the association between child age categories and treatment failure, the older children had a lower risk of treatment failure (Table 3). While hypoxia and age categories up to 23 months remained as independent predictors, the oldest age group and any danger sign were no longer significant as indicators of treatment failure in models 2 and 3 (Table 3). Gender, being breastfed, stunted, having high fever, elevated CRP, viruses in the nasopharynx, and zinc supplementation were not significantly associated with treatment failure.

**Table 3:** Predictors of Treatment failure of illness episode in children 2–35 months hospitalized with WHO defined Severe Pneumonia

Variables	Crude Odds Ratio*	Adjusted Odds ratio* (number of observations per model)		
**Age in months**	0.93 (0.90, 0.96) <0.001			
**Age categories**		**Model 1 (590)**	**Model 2 (455)**	**Model 3 (454)**
• 2–6 months	1.00	1.00	1.00	1.00
• 7–11 months	0.67 (0.44, 1.01) 0.058	0.61 (0.40, 0.94) 0.024	0.56 (0.34, 0.91) 0.019	0.55 (0.34, 0.90) 0.018
• 12–23 months	0.37 (0.21, 0.64) <0.001	0.33 (0.19, 0.58) <0.001	0.27 (0.13, 0.55) <0.001	0.27 (0.13, 0.55) <0.001
• 24–35months	0.22 (0.05, 0.99) 0.049	0.18 (0.04, 0.83) 0.028	0.31 (0.06, 1.54) 0.153	0.34 (0.07, 1.72) 0.194
**Gender**	0.83 (0.59,1.18) 0.296			
**Breastfed**	0.67 (0.28,1.61) 0.370			
Wasting (594) (< -2 Weight for height/length)	1.49 (1.02, 2.16) 0.038			
Stunting (598) (< -2 Height/Length for age)	1.25 (0.69, 2.27) 0.457			
**Hypoxia** (SpO2 < 90%)	1.91 (1.33, 2.74) <0.001	2.00 (1.36, 2.93) <0.001	1.80 (1.15, 2.80) 0.010	1.81 (1.16, 2.83) 0.009
**Febrile** (Axillary temperature > 38.5°C)	0.94 (0.59, 1.51) 0.808			
**Any danger sign**	1.65 (1.18, 2.32) 0.004	1.44 (1.01, 2.07) 0.045		
**Radiographic pneumonia**	2.22 (1.49, 3.31) < 0.001		2.09 (1.37, 3.19) 0.001	2.12 (1.39, 3.24) 0.001
**C-Reactive Protein (CRP) > 40 mg/L**	1.15 (0.79, 1.67) 0.472			
**Nasopharyngeal aspirate positive for virus**				
• Respiratory syncytial virus (RSV)	1.47 (0.91, 2.38) 0.115			
• Influenza A	0.31, 1.84) 0.533			
• Influenza B	0.37, 2.76) 0.989			
• Parainfluenza type 1 (PIV 1)	0.18, 1.37) 0.178			
• Parainfluenza type 2 (PIV 2)	0.20, 7.44) 0.819			
• Parainfluenza type 3 (PIV 3)	0.12, 1.06) 0.063			
• Human metapneumovirus (hMPV)	2.34 (0.62, 8.81) 0.209			
**Supplemented with zinc**	0.83 (0.60, 1.17) 0.291			

*Odds ratios (95% CI) and P-value calculated using Logistic Regression. Odds ratios > 1 indicates increased odds of treatment failure.

Results of multiple regressions with P-value >0.05 not shown in the table with the exception of age analyzed as a categorical variable.

Model 1 (Clinical variables): Adjusted for gender, breastfed, wasting, febrile and treatment with zinc.

Model 2 (Clinical + Radiographic pneumonia): Adjusted for gender, breastfed, wasting, febrile, any danger sign and treatment with zinc.

Model 3 (All variables): Adjusted for gender, breastfed, wasting, febrile, any danger sign, CRP, nasopharyngeal aspirate positive for RSV, PIV 1, 2 and 3, influenza A and B, hMPV and treatment with zinc.

We repeated our regression analysis excluding hypoxia as a covariate in all three models and found very little change in the hazard and odds ratios of predictors, age and radiographic pneumonia identified earlier (data not shown). However, in the absence of hypoxia as a covariate, presence of at least one danger sign was a significant predictor of treatment failure in all models (data not shown). We also repeated the analyses using lower cut offs to define hypoxemia i.e. SpO2 of 88% and 85% but the regression coefficients were only marginally altered (data not shown).

We explored possible interactions between independent variables using interaction terms (age x breastfeeding, age x wasting and age x any danger sign). Age did not modify the effect of breastfeeding, wasting or any danger sign in any of the models.

## Discussion

We report findings from secondary analyses of data from a clinical trial assessing the efficacy of zinc in childhood pneumonia. In this large prospective study of hospitalized young Nepalese children with acute severe pneumonia, median time till recovery of 2 days and treatment failure in 35%, independent predictors for both outcomes were younger age, radiographic consolidation and hypoxia on admission. In addition, wasting and any danger sign on admission was associated with prolonged duration of illness. With the exception of detecting PIV 1 from the nasopharynx, which signaled earlier recovery, presence of other viruses and raised CRP levels were not associated with either outcome. As this was a clinical trial on the efficacy of zinc in children with severe pneumonia, we also explored the possibility of differences in the outcomes between those who received the drug versus those who did not, and found no such interaction.

While several studies report on predictors of death [12–15], we were unable to find any exploring clinical risk factors for duration of illness of a severe pneumonia episode in young children. In our study only 4 children died in the hospital, and mortality was not an outcome. Hypoxia, a significant predictor of death in most of the studies [12,13,15] was an independent predictor of time till resolution of illness. While studies have identified younger children to be at a higher risk of death [14,15], we found slower time till recovery with decreasing age (Table 2). To our knowledge, this is the first study to identify age as an independent predictor of the duration of severe pneumonia.

The proportions with treatment failure in large multicenter clinical trials conducted in hospitalized children with severe pneumonia were 13.6% [16] and 20% [17]. These estimates are different from our study (35%) and may partly be due to the differences in outcome definition. Similar to the present study, hypoxia on admission was identified as an independent predictor of treatment failure in both studies. The trends for age as a predictor were similar in our study and the study by Fu. *et al*. [18], with younger children having a higher risk of treatment failure.

We found hypoxia to be a predictor of both treatment failure and illness duration in our study. With hypoxia removed as a covariate, presence of any danger sign was a significant predictor of treatment failure in all three models. The variables hypoxia and any danger sign were correlated, which is expected as both are known indicators of severe pneumonia. Clinical predictors may not be easily recognized whereas measurement of oxygen saturation with a pulse oximeter, when performed correctly, to document hypoxia seems to be a more reliable and objective sign to assess severity. Pulse oximetry, the ‘fifth pediatric vital sign’ [19] supplemented by treatment with oxygen was found to be a feasible and cost effective intervention for childhood pneumonia with 35% reduction in mortality in Papua New Guinea [20]. Unfortunately even though pulse oximeters and oxygen are available, they are underutilized by health workers in developing countries [21,22]. Pulse oximetry to document hypoxia in community acquired pneumonia is not only endorsed by professional societies in developed countries [23,24] but also stated in recent WHO guidelines[6] and our findings support these recommendations.

We identified one study in India [25] reporting lobar consolidation as a predictor of longer stay in hospital but this was based on unadjusted analysis and therefore not comparable to the results of our study. The two studies that report an association between radiographic consolidation and mortality in hospitalized children with severe pneumonia have conflicting results. While Lupisan *et al*. [14] found dense infiltrates to be an independent predictor, Reed *et al*. [12] report that alveolar consolidation on multiple regression analysis was not significant. Primary end point consolidation was an independent predictor of treatment failure at 48 hours of hospitalization especially among penicillin recipients (RR 3.58 95% CI: 1.47, 8.75) in the study by Patel *et al*. [17], a finding very similar to what we found. In our study, chest radiograph was done to screen for exclusion criteria and had no role in the initial diagnosis and management of enrolled patients. We found that addition of radiographic pneumonia to the analyses (Models 2 and 3) did not substantially change the estimates of independent clinical predictors of both outcomes (Tables 2 and 3). In resource poor settings, diagnosis of severe pneumonia relies heavily on clinical signs. An initial chest radiograph in uncomplicated but severe community acquired pneumonia would not change initial management with empirical antibiotic therapy in such settings. Our findings support the WHO guidelines [6] which state that radiographs in severe pneumonia should be done only when possible.

We report findings from a randomized clinical trial [7] conducted under controlled settings. Our findings may therefore not be directly applicable to other hospitals caring for children in developing countries. We used previous WHO guidelines [8]requiring only lower chest indrawing in addition to symptoms to define severe pneumonia as inclusion criteria. Although we took measures to screen out children with reactive airways, we may have included those with pneumonia that could have been treated as outpatients. While recruiting, we excluded very sick children with pneumonia and probably missed those with other signs of severe pneumonia in the absence of LCI and this may also have affected our observed associations. While we were able to detect several predictors for duration and treatment failure, for many of the selected independent variables such as fever and breastfeeding, the power to detect any significant association with the outcomes was low. It should be noted that the study was designed to measure the effect of zinc supplementation during pneumonia and not to identify these predictors.

This large study on children aged between 2–35 months, with acute severe pneumonia found younger age, hypoxia on admission and radiographic pneumonia to independently predict both time to resolution and treatment failure. As it is the only study till date to report on predictors of illness duration we believe our findings to be relevant, not only to characterize but also manage a severe pneumonia episode in hospitalized children from resource poor settings. Our results indicate that a chest radiograph is not always needed but the detection and treatment of hypoxia is a crucial step in the management of children admitted to health care settings with pneumonia.

## Supporting Information

S1 Dataset (XLSX)
